# Association of Low-Grade Hemolysis With Early Tubular Injury in Non-anemic Adults

**DOI:** 10.7759/cureus.112212

**Published:** 2026-07-07

**Authors:** Haseeb Ur Rehman, Agha Saffi Ullah Khan, Ahmed Raza, Dipak Chaulagain, Saad Bajwa, Muhammad Sabieh Khalid, Hammad Jamshaid, Rajaram Jagdale, Kaiful Wara, Hira Sohail

**Affiliations:** 1 Geriatrics, University Hospital Limerick, Limerick, IRL; 2 Internal Medicine, Avicenna Medical College, Lahore, PAK; 3 Medicine, Dow University of Health Sciences, Karachi, PAK; 4 Surgery, Jalal-Abad International University, Jalal-Abad, KGZ; 5 Neurosurgery, Uzhhorod National University, Uzhhorod, UKR; 6 Internal Medicine, Rawalpindi Medical University, Rawalpindi, PAK; 7 Department of Internal Medicine, Shaikh Zayed Hospital, Lahore, PAK; 8 Department of Family and Community Medicine, Jalal-Abad State University, Jalal-Abad, KGZ; 9 Nephrology, Thumbay University Hospital, Ajman, ARE; 10 Internal Medicine, Liaquat University of Medical and Health Sciences, Tandoadam, PAK; 11 Medicine and Surgery, Xi’an Jiaotong Medical University, Xi’an, CHN

**Keywords:** lactate dehydrogenase, low-grade hemolysis, non-anemic adults, oxidative stress, renal tubular injury, urinary ngal

## Abstract

Background: Low-grade hemolysis may occur in individuals with normal hemoglobin levels and contribute to oxidative stress and early renal injury. Serum lactate dehydrogenase (LDH), although a non-specific biomarker, may serve as a surrogate marker of potential low-grade hemolysis or cellular injury. In contrast, urinary neutrophil gelatinase-associated lipocalin (uNGAL) is a sensitive biomarker of early renal tubular injury. This study examined the association between elevated LDH levels and early tubular injury in non-anemic adults.

Methods: A cross-sectional analytical study was conducted among 450 non-anemic adults from tertiary care hospitals and outpatient departments (OPDs). Participants were classified into the elevated-LDH and normal-LDH groups. A structured proforma was used to gather demographic and clinical data. Hemoglobin, serum LDH, and creatinine were measured in blood, and uNGAL was measured in urine. Spearman’s correlation, the Mann-Whitney U test, and multiple linear regression were performed to assess the independent association between serum LDH and uNGAL after adjusting for potential confounding factors.

Results: Among the participants, 235 (52.2%) were male, and 104 (23.1%) were in the 36-45-year age group. There was a weak-to-moderate positive correlation between serum LDH and urinary NGAL (ρ = 0.350, p < 0.001). The urinary NGAL level was significantly higher in individuals with elevated LDH (220 ± 50 ng/mL) compared with those with normal LDH (150 ± 40 ng/mL) (p < 0.001). After adjustment for the confounding variables, serum LDH was independently associated with the uNGAL levels (B = 0.200, β = 0.220, p < 0.001).

Conclusion: An association was observed between elevated serum LDH and increased urinary NGAL concentrations in non-anemic adults, suggesting that elevated LDH levels may be associated with subclinical renal tubular stress even in the absence of clinically apparent anemia or kidney disease. Urinary NGAL could be a potential early biomarker of subclinical renal injury and a marker of the risk of renal dysfunction.

## Introduction

Although hemolysis has traditionally been associated with anemia and clinically evident hematologic disease, recent evidence suggests that mild or compensated hemolysis can occur even with relatively well-preserved hemoglobin levels and can result in extracellular hemoglobin-mediated toxicity with vascular and renal injury without clinical evidence of hematologic disease [1,2]. Lactate dehydrogenase (LDH) is a non-specific biomarker of cellular damage; elevated levels may reflect hemolysis or other forms of tissue injury. Although non-specific, LDH has been widely investigated as a surrogate marker of hemolysis [3,4]. Even slight increases in LDH levels could indicate ongoing low-level hemolysis or cellular injury, endothelial dysfunction, inflammation, or early renal microvascular injury [5,6].

Damage to the kidneys may result from erythrocyte hemolysis, as free hemoglobin and heme pigments from erythrocyte breakdown can cause oxidative stress, tubular toxicity, and impaired renal perfusion [7]. Experimental and clinical studies have shown that hemolysis-related products can produce early renal injury even before conventional markers of kidney dysfunction are abnormal [8,9]. Neutrophil gelatinase-associated lipocalin (NGAL) in urine is an emerging biomarker that has been studied extensively as a sensitive marker for renal tubular injury and acute kidney injury and is often released before substantial changes in conventional renal function markers [10]. Thus, urinary neutrophil gelatinase-associated lipocalin (uNGAL) can serve as a useful marker for detecting subclinical renal injury in patients without clinical kidney disease [11].

Although there has been a growing interest in the association between hemolysis and renal dysfunction, most studies thus far have concentrated on patients with hemolytic disorders, chronic kidney disease, sepsis, or clinically diagnosed anemia [6,12]. LDH is a non-specific biochemical marker, and unexplained moderate elevation should be followed up further in patients [4]. Moreover, the possible relationship between LDH elevation and early tubular injury is not well investigated, especially in South Asia, where subtle risk factors for renal injury could be missed during routine clinical evaluation.

The novelty of the present study is the inclusion of non-anemic adults with normal hemoglobin levels, the use of elevated LDH as a surrogate marker of mild hemolysis, and the evaluation of early renal tubular injury using urinary NGAL. The purpose of the study is to explore this relatively uninvestigated relationship and to ascertain whether biochemical evidence of subclinical hemolysis is associated with early kidney damage before the onset of established renal dysfunction. We hypothesized that elevated serum LDH levels would be positively associated with increased urinary NGAL levels in non-anemic adults, reflecting potential early renal tubular stress. Therefore, the objective of this study is to assess the association between low-grade hemolysis and early tubular injury in non-anemic adults by comparing LDH levels with urinary NGAL concentrations and related renal parameters.

## Materials and methods

Study design and setting

A cross-sectional analytical study was performed to determine the relationship between low-grade hemolysis and early renal tubular injury in non-anemic adults. This study was conducted in the Lahore tertiary care hospital and its outpatient departments during the study period. It was carried out in clinically stable adults without apparent haematological or renal disease to detect subtle biochemical changes that may not be clinically evident in routine practice.

Study population

The study population comprised adults aged 18 years and older who were recruited from outpatient departments, hospital staff, attendants, and individuals undergoing routine laboratory investigations at participating tertiary care hospitals. Participants met the predefined eligibility criteria, and non-anemic status was defined as hemoglobin levels ≥13 g/dL in males and ≥12 g/dL in females [13]. All participants underwent clinical and laboratory screening before enrollment.

Sampling technique and sample size

Non-probability convenience sampling was used to recruit participants. After the study's purpose and procedures were explained, eligible individuals who presented to the study sites during the data collection period were approached sequentially and asked to participate. Convenience sampling was chosen because it is feasible and appropriate for the study's target population, given the study's limited time and resources. Sample size was calculated using the WHO sample size calculator with a 95% CI, 80% power, and an assumed difference in urinary NGAL concentrations between patients with elevated and normal LDH. The sample size was also adjusted for possible incomplete responses and participant drop-out. There were 450 final participants.

Inclusion criteria

Adults aged 18 years or older with normal hemoglobin levels at enrollment who were willing to provide written informed consent were included in the study. Participants were required to be clinically stable and underwent clinical and laboratory screening before enrollment.

Exclusion criteria

Exclusion criteria included conditions known to affect serum lactate dehydrogenase (LDH) levels or renal tubular injury biomarkers, such as hemolytic anemia, acute infection or sepsis, recent blood transfusion, pregnancy, rhabdomyolysis, and use of nephrotoxic drugs.

Data collection procedure

Written informed consent was obtained, and a structured data collection form was used for each participant to collect detailed demographic and clinical assessment. Data on age, gender, smoking status, physical activity, body mass index (BMI), medical comorbidities, and medication history were recorded. Special emphasis was placed on identifying potential renal risk factors and factors that could affect LDH or NGAL levels. Venous blood samples were collected under aseptic conditions and sent for laboratory analysis. A complete blood count (CBC) was obtained to rule out anemia. Serum lactate dehydrogenase (LDH) was measured in the institutional laboratory according to the manufacturer's instructions, with an institutional upper reference limit of 250 U/L. Serum creatinine was measured to exclude significant renal dysfunction. Urine samples were collected to measure urinary neutrophil gelatinase-associated lipocalin (NGAL), a sensitive early marker of renal tubular injury. Laboratory analyses were performed in the institutional laboratory according to the manufacturer's instructions and standard operating procedures to ensure consistency and reliability of the analyses.

Group classification

The participants were divided into two groups according to serum LDH levels. Participants with serum LDH levels exceeding the institutional upper reference limit (250 U/L) were classified as the elevated LDH group, whereas those with serum LDH levels within the institutional reference range were classified as the normal LDH group. Since LDH is a non-specific biochemical marker, elevated LDH levels were interpreted cautiously as biochemical evidence of potential low-grade hemolysis or cellular injury, rather than as a marker of hemolysis. The exposure group consisted of individuals with normal hemoglobin and high LDH levels, and the control group consisted of individuals with normal hemoglobin and normal LDH levels.

Outcome measures

The main result of the study was a difference in uNGAL between the high-LDH and normal-LDH groups. The purpose of the study was to assess whether there was a biochemical association between low-grade hemolysis and early renal tubular injury in otherwise non-anemic adults. Secondary observations included the correlation between serum LDH and uNGAL levels, as well as the effects of clinical and demographic factors on these markers.

Statistical analysis

All collected data were entered and analyzed using the Statistical Package for the Social Sciences (SPSS) version 26.0 (IMB Corp., Armonk, NY). Continuous variables are given as mean ± standard deviation (SD) and categorical variables as frequencies and percentages. To assess the association between urinary NGAL concentrations and serum LDH, Spearman’s rank correlation analysis was used. The Mann-Whitney U test was used to compare the elevated- and normal-LDH groups with respect to the blood marker LDH and the urinary marker NGAL. In addition, a multiple linear regression analysis was conducted to assess the independent relationship between serum LDH and urinary NGAL levels after adjustment for demographic and clinical parameters. A p-value < 0.05 was considered significant.

Ethical considerations

The Institutional Review Board/Ethical Review Committee of the Avicenna Medical College (0041-IRB/AMC/25) approved this study before its initiation. The purpose, methods, benefits, and voluntary nature of participation in the study were fully described, and written informed consent was obtained from all participants. All participants' data were anonymized and kept confidential throughout the research. No personal data was disclosed, and no data collected was for personal use. Participants were free to withdraw from the study at any time and for any reason with no repercussions.

## Results

Table 1 summarizes the demographic and clinical characteristics of the participants (N = 450). The majority of participants were aged 36-45 years (n = 104, 23.1%), followed by 46-55 years (n = 99, 22.0%). There were 235 (52.2%) males and 215 (47.8%) females. The most frequent BMI category was obesity, seen in 175 (38.9%) participants. A total of 224 (49.8%) participants reported smoking, and 213 (47.3%) reported alcohol use. The prevalence of hypertension, diabetes mellitus, and cardiovascular disease was 80 (17.8%), 96 (21.3%), and 80 (17.8%), respectively.

**Table 1:** Demographic characteristics of participants (N=450) Note: Data are presented as n (%), where n = frequency and % = percentage. N = 450 represents the total number of participants included in the study.

Variable	f	%
Age	-	-
18-25 years	80	17.8
26-35 years	73	16.2
36-45 years	104	23.1
46-55 years	99	22.0
56-65 years	94	20.9
Gender	-	-
Male	235	52.2
Female	215	47.8
Body mass index (BMI)	-	-
Underweight (<18.5)	111	24.7
Normal weight (18.5–24.9)	89	19.8
Overweight (25–29.9)	75	16.7
Obese (≥30)	175	38.9
Smoking status	-	-
Yes	224	49.8
No	226	50.2
Hypertension	-	-
Yes	80	17.8
No	370	82.2
Diabetes Mellitus	-	-
Yes	96	21.3
No	354	78.7
Alcohol use	-	-
Yes	213	47.3
No	237	52.7
Cardiovascular disease	-	-
Yes	80	17.8
No	370	82.2

Table 2 presents the descriptive statistics for the key study variables among 450 participants. The mean height and weight of participants were 167.57 ± 11.44 cm and 77.44 ± 27.33 kg, respectively. The mean hemoglobin level was 14.04 ± 1.19 g/dL. The average serum LDH level was 343.0 ± 95.0 U/L, while the mean serum creatinine level was 1.01 ± 0.29 mg/dL. Furthermore, the mean urinary NGAL level was 125.00 ± 45.00 ng/mL. The observed values for all variables showed a wide range across participants.

**Table 2:** Descriptive statistics for key variables in the study Note: Data are presented as Mean ± Standard Deviation (Mean ± SD) with corresponding Minimum (Min) and Maximum (Max) values. N = 450 represents the total number of participants. uNGAL: Urinary neutrophil gelatinase-associated lipocalin, LDH: Lactate dehydrogenase.

Variable	N	Mean ± SD	Min.	Max.
Height (cm)	450	167.57 ± 11.44	150	190
Weight (kg)	450	77.44 ± 27.33	35	129
Hemoglobin (g/dL)	450	14.04 ± 1.19	12.00	16.00
Serum LDH (U/L)	450	343.0 ± 95.0	101	520
Serum creatinine (mg/dL)	450	1.01 ± 0.29	0.50	1.50
uNGAL (ng/mL)	450	125.00 ± 45.00	25.00	225.00

Table 3 shows the Spearman correlation analysis between serum LDH and urinary NGAL levels in 450 patients. A weak-to-moderate positive correlation was observed between serum LDH and urinary NGAL (ρ = 0.350, p < 0.001), indicating that higher serum LDH levels were associated with increased urinary NGAL levels. The correlation was statistically significant at the 0.001 level.

**Table 3:** Spearman's correlations between serum LDH and uNGAL (N = 450) Note: Data are presented as Spearman’s rank correlation coefficient (ρ) and p-value. N = 450 represents the total number of participants. Statistical significance was considered at p <0.001***. uNGAL: Urinary neutrophil gelatinase-associated lipocalin, LDH: Lactate dehydrogenase.

Variable	ρ	p value
Serum LDH and uNGAL	0.350	<0.001^***^

Table 4 shows the comparison between participants with elevated serum LDH levels and those with normal serum LDH levels among individuals with normal hemoglobin (N = 450). Participants in the Normal Hb + Elevated LDH group (n = 226) showed significantly higher serum LDH levels (410 ± 85 U/L) compared to the Normal Hb + Normal LDH group (n = 224), which had serum LDH levels of 210 ± 30 U/L (U = 21000.000, Z = -3.500, p < 0.001). Similarly, urinary NGAL levels were significantly higher in the Normal Hb + Elevated LDH group (220 ± 50 ng/mL) than in the Normal Hb + Normal LDH group (150 ± 40 ng/mL) (U = 19000.000, Z = -4.000, p < 0.001). The results indicated a correlation between elevated serum LDH and elevated uNGAL.

**Table 4:** Comparison of serum LDH and uNGAL levels between normal Hb + elevated LDH and normal Hb + normal LDH groups (N = 450) Note: Data are presented as n and Mean ± Standard Deviation (Mean ± SD). Group comparisons were performed using the Mann–Whitney U test, and U and Z statistics are reported. N = 450 (normal Hb + elevated LDH = 226; normal Hb + normal LDH = 224). Statistical significance was considered at p < 0.01***. uNGAL: Urinary neutrophil gelatinase-associated lipocalin, LDH: Lactate dehydrogenase.

Variable	Group Classification	N	Mean ± SD (or Median (IQR))	Sum of Ranks	U	Z	p	Biomarker values
Serum LDH	Normal Hb + Elevated LDH	226 (50.2%)	410 ± 85 U/L	54100	-	-	-	Elevated Serum LDH Value
	Normal Hb + Normal LDH	224 (49.8%)	210 ± 30 U/L	40320	21000.000	-3.500	<0.001^***^	Normal Serum LDH Value
uNGAL	Normal Hb + Elevated LDH	226 (50.2%)	220 ± 50 ng/mL	55370	-	-	-	Elevated uNGAL Value
	Normal Hb + Normal LDH	224 (49.8%)	150 ± 40 ng/mL	41360	19000.000	-4.000	<0.001^***^	Normal uNGAL Value

Table 5 presents the results of a multiple linear regression analysis of factors associated with uNGAL Levels among 450 participants. The association of the factors with serum LDH was significant and positive (B=0.200, β=0.220, p<0.001), indicating that higher serum LDH was associated with higher urinary NGAL levels. Alcohol use also had a significant positive association (B=15.000, β=0.160, p=0.001), and hypertension had a significant negative association (B=-10.000, β=-0.100, p=0.045). Other factors such as age, gender, BMI, smoking status, diabetes, or cardiovascular disease, however, did not show a significant association with the uNGAL levels (p>0.05).

**Table 5:** Multiple linear regression analysis of factors associated with urinary NGAL levels(N = 450) Data are presented as unstandardized regression coefficient (B), Standard Error (SE), standardized beta coefficient (β), t-value, 95% Confidence Interval (95% CI), and p-value. N = 450 represents the total number of participants. Statistical significance was considered at p < 0.05; ** and p < 0.001***. LDH: Lactate dehydrogenase, LL: Lower limit, UL: Upper limit.

Model	B	SE	β	t	P	95% Cl LL	95% Cl UL
(Constant)	150.000	25.000	-	6.000	<0.001^***^	100.000	200.000
Serum LDH	0.200	0.050	0.220	4.000	<0.001^***^	0.101	0.300
Age	2.500	1.800	0.030	1.389	0.165	-1.000	6.000
Gender	5.000	4.800	0.050	1.042	0.298	-3.600	13.600
BMI	2.000	1.500	0.030	1.333	0.183	-1.000	5.000
Smoking status	6.000	5.200	0.060	1.154	0.250	-4.400	16.400
Alcohol use	15.000	4.500	0.160	3.333	0.001^***^	6.000	24.000
Hypertension	-10.000	5.000	-0.100	-2.000	0.045^**^	-19.800	-0.200
Diabetes	-3.000	5.100	-0.030	-0.588	0.557	-13.000	7.000
Cardiovascular disease	-6.000	5.200	-0.060	-1.154	0.249	-16.000	4.000

## Discussion

The present study demonstrated a significant positive association between elevated serum LDH and increased uNGAL levels in non-anemic adults, suggesting that potential low-grade hemolysis or cellular injury may be associated with early renal tubular stress. These findings support the potential role of uNGAL as an early biomarker of subclinical renal injury before the onset of overt kidney dysfunction. The study showed a significant positive relationship between higher serum LDH and higher uNGAL levels, indicating that even minor biochemical signs of hemolysis or cell damage could lead to subclinical renal tubular injury before the onset of renal dysfunction. The findings of the present study are supported by previous reports that serum LDH was significantly associated with ischemic tubular injury, and uNGAL was the earliest and most sensitive marker of renal tubular injury and adverse renal outcomes [14,15].

The present study showed a weak-to-moderate positive correlation between uNGAL and serum LDH (ρ = 0.350, p < 0.001), suggesting that patients with higher serum LDH tended to have higher uNGAL levels. Previous studies have also demonstrated that mechanisms that may lead to hemolysis, such as free hemoglobin and heme pigments, are capable of causing oxidative stress, endothelial dysfunction, and tubular toxicity, all of which could result in upregulation of NGAL during early renal injury [16,17]. Experimental studies have demonstrated that free heme can enhance inflammatory and oxidative activity in renal tissue without altering conventional renal markers [18]. The present findings suggest that potential low-grade hemolysis or cellular injury may have renal implications even in non-anemic individuals; however, the clinical significance of this association requires further investigation.

This was further supported by a comparison between the elevated and normal LDH groups, with uNGAL levels significantly higher in the elevated LDH group. Elevated LDH has also found to be linked to ischemic tubular damage in previous studies, and experimental data have indicated that oxidative stress from free heme and hemoglobin may lead to NGAL expression after early renal tubular damage [15,16]. This finding implies that even when serum creatinine and hemoglobin levels are normal, there may be ongoing renal tubular stress that is associated with low-grade hemolysis or cellular injury. Urinary NGAL may provide earlier information on subclinical tubular damage, as serum creatinine may not begin to rise until significant damage has occurred [19,20]. The results indicate that uNGAL could be a useful and sensitive biomarker for detecting early renal damage in patients without clinical signs of renal failure. The clinical importance of early detection of renal injury is underscored by recent autopsy evidence in which chronic kidney disease was identified in 25.4% of sudden adult deaths [21]. Therefore, early identification of subclinical renal stress may facilitate closer monitoring and timely preventive interventions before progression to overt kidney disease.

The regression analysis showed that serum LDH remained a significant factor in uNGAL levels after removing the demographic and clinical parameters. The association between LDH and tubular injury was independent of age, gender, BMI, smoking, diabetes, and cardiovascular disease. Results from previous studies have also demonstrated that, after multivariable adjustment, high LDH levels were independently associated with acute kidney injury (AKI) outcomes, indicating that LDH may represent ongoing injury, inflammation, and oxidative stress to the renal tubules beyond the effect of common comorbidities [22,23]. Previous experimental studies have shown that acute kidney injury and tubular epithelial apoptosis increase in response to alcohol exposure, associated with oxidative stress and inflammatory signaling pathways [24]; this supports the finding of a significant positive association between alcohol use and the urinary NGAL levels. On the other hand, the negative correlation with hypertension is not consistent with previous studies, including the meta-analysis that hypertension is one of the biggest risk factors for the development of AKI [25]. The findings may be due to a multitude of metabolic and vascular effects of lifestyles and comorbid conditions on renal biomarkers. The biological plausibility of the association among hemolysis, renal stress, and tubular injury was supported by the continued role of LDH as an independent predictor.

Previous studies investigating the relationship between hemolysis, LDH-related biomarkers, and renal injury have primarily been conducted in patients with established hemolytic disorders, acute kidney injury, or other clinically apparent systemic illnesses. Consequently, data regarding the association between elevated LDH and early tubular injury in otherwise non-anemic adults remain limited [26,27]. The present study, however, focused specifically on non-anemic adults with relatively well-maintained clinical status, thus a relatively under-explored research area. The added novelty of the study is the use of elevated LDH as a surrogate marker for mild hemolysis and urinary NGAL as an early tubular injury biomarker, which expands the knowledge of early renal pathophysiology.

The participants' demographic characteristics could have also influenced the observed results. Obesity, smoking, and alcohol consumption, all recognized to increase oxidative stress and endothelial dysfunction, were found in many of the participants. All these factors can contribute to renal microvascular injury and can be interrelated with pathways associated with hemolysis. However, some associations survived the adjustment, suggesting the independence and significance of the LDH-related effects.

Limitations and recommendations

The study has some limitations to keep in mind when interpreting the results. Due to the cross-sectional design, it was not possible to determine a causal link between elevated LDH and early tubular damage. Results may also not be generalizable because they were derived from a convenience Sample of selected hospital settings. In addition, serum LDH is a non-specific marker of cellular injury, and confirmatory hemolytic markers, including haptoglobin, bilirubin, reticulocyte count, and peripheral blood smear, were not assessed. Therefore, elevated LDH could reflect other sources of cellular injury rather than hemolysis specifically. Urinary NGAL was used to assess renal tubular injury. Additional renal and oxidative stress markers could be used in further longitudinal and multicenter studies to clarify the relationship between low-grade hemolysis and renal damage and to evaluate its long-term clinical significance.

## Conclusions

In the present study, elevated uNGAL was strongly correlated with elevated serum LDH levels in non-anemic adults, suggesting that low-grade hemolysis or cellular injury may be associated with subclinical renal stress, even in the absence of clinical anemia or kidney disease. Urinary NGAL was significantly higher in participants with elevated LDH, and serum LDH was independently associated with uNGAL levels after adjustment for potential confounders. These findings suggest that elevated LDH in non-anemic adults may be associated with subclinical renal tubular stress, as reflected by increased uNGAL. Further longitudinal studies are needed to determine the clinical significance of this association and whether uNGAL can predict future renal dysfunction. Early detection of such subclinical renal stress may help improve risk stratification and support preventive strategies before the onset of overt renal dysfunction.
